# Identification of Potential Oral Microbial Biomarkers for the Diagnosis of Periodontitis

**DOI:** 10.3390/jcm9051549

**Published:** 2020-05-20

**Authors:** Hee Sam Na, Si Yeong Kim, Hyejung Han, Hyun-Joo Kim, Ju-Youn Lee, Jae-Hyung Lee, Jin Chung

**Affiliations:** 1 Department of Oral Microbiology, School of Dentistry, Pusan National University, Busandaehak-ro 49, Mulgeum-eup, Yangsan-si, Gyeongsangnam-do 50612, Korea; heesamy@pusan.ac.kr (H.S.N.); gji0307@naver.com (S.Y.K.); rosso2451@naver.com (H.H.); 2 Oral Genomics Research Center, School of Dentistry, Pusan National University, Busandaehak-ro 49, Mulgeum-eup, Yangsan-si, Gyeongsangnam-do 50612, Korea; 3 Department of Periodontology, Dental and Life Science Institute, School of Dentistry, Pusan National University, Busandaehak-ro 49, Mulgeum-eup, Yangsan-si, Gyeongsangnam-do 50612, Korea; zoo0316@naver.com (H.-J.K.); heroine@pusan.ac.kr (J.-Y.L.); 4 Department of Periodontology and Dental Research Institute, School of Dentistry, Pusan National University, Busandaehak-ro 49, Mulgeum-eup, Yangsan-si, Gyeongsangnam-do 50612, Korea; 5 Department of Oral Microbiology, School of Dentistry, Kyung Hee University, 26 Kyungheedae-ro, Dongdaemun-gu, Seoul 02447, Korea; 6 Department of Life and Nanopharmaceutical Sciences, Kyung Hee University, 26 Kyungheedae-ro, Dongdaemun-gu, Seoul 02447, Korea

**Keywords:** oral bacteria, biomarkers, bioinformatics, microbiome, periodontal disease(s)/periodontitis

## Abstract

Periodontitis is a chronic and multifactorial inflammatory disease that can lead to tooth loss. At present, the diagnosis for periodontitis is primarily based on clinical examination and radiographic parameters. Detecting the periodontal pathogens at the subgingival plaque requires skilled professionals to collect samples. Periodontal pathogens are also detected on various mucous membranes in patients with periodontitis. In this study, we characterized the oral microbiome profiles from buccal mucosa and supragingival space in a total of 272 healthy subjects as a control group, and periodontitis patients as a disease group. We identified 13 phyla, 193 genera, and 527 species and determined periodontitis-associated taxa. *Porphyromonas gingivalis*, *Tannerella forsythia*, *Treponema denticolar*, *Filifactor alocis*, *Porphyromonas endodontalis*, *Fretibacterium fastiosum* and *Peptostreptococcus* species were significantly increased in both the buccal mucosa and the supragingival space in periodontitis patients. The identified eight periodontitis-associated bacterial species were clinically validated in an independent cohort. We generated the prediction model based on the oral microbiome profiles using five machine learning algorithms, and validated its capability in predicting the status of patients with periodontitis. The results showed that the oral microbiome profiles from buccal mucosa and supragingival space can represent the microbial composition of subgingival plaque and further be utilized to identify potential microbial biomarkers for the diagnosis of periodontitis. Besides, bacterial community interaction network analysis found distinct patterns associated with dysbiosis in periodontitis. In summary, we have identified oral bacterial species from buccal and supragingival sites which can predict subgingival bacterial composition and can be used for early diagnosis of periodontitis. Therefore, our study provides an important basis for developing easy and noninvasive methods to diagnose and monitor periodontitis.

## 1. Introduction

Periodontitis is a multifactorial and inflammatory disease that affects about 30%–35% of the global population and about 70% of individuals 65 years of age and older. If untreated, periodontitis results in periodontal tissue destruction and loss of teeth eventually. Moreover, it increases with age and is closely associated with systemic diseases, such as cardiovascular disease, diabetes, and even Alzheimer’s disease, etc. Periodontal disease is an inflammatory disease caused by host immune responses to bacterial infections. Bacteria in the periodontal pocket can easily access the inflamed epithelium and travel to other body sites via the bloodstream [1]. Thus, the inflammations caused by the periodontitis do not only remain in the oral cavity, but could be spread throughout the body. Three main well-known mechanisms: metastatic infection, metastatic injury, and metastatic inflammation explain how the periodontitis is connected with the systemic diseases [2]. Recently, it has been reported that the treatment of the periodontitis improves the systemic diseases [3] and saliva could be used as a biomarker for one of the systemic disease, cystic fibrosis [4]. As described above, the traveling bacteria could cause the systemic diseases and worsen the systemic diseases especially for the patients with underlying diseases. Therefore, early diagnosis and monitoring of periodontitis can be a good practice for the prevention of the systemic disease as well as the periodontal disease. At present, the diagnosis of periodontitis is primarily based on clinical examination and radiographic parameters [5] that appear only when periodontitis progresses. Unfortunately, there is no microbiologic or histopathologic assessment for clinicians to support the diagnosis. Commensal microbiota in healthy oral cavity live in harmonious symbiotic relationship, while the initiation of periodontitis is closely associated with a dramatic change of microbial community referred to as dysbiosis [6]. Therefore, the development of peritonitis-associated bacterial panels could provide the early diagnosis for the periodontitis and the prognosis of the disease after treatments.

Gram-negative *Porphyromonas gingivalis*, *Treponema denticola*, and *Tannerella forsythia* were frequently isolated from dental plaques in periodontal patients and were considered specific pathogens of periodontal disease [7]. A strong correlation between the proportions of several cultivable bacteria and periodontal disease has been reported [8,9,10]. More recently, culture-independent molecular techniques were applied to identify oral bacteria more abundant in periodontal lesions [11,12]. During the past 10 years, the next-generation sequencing (NGS) of bacterial 16S rRNA (ribosomal RNA) gene has provided us with a deeper understanding of the complexity of the microbiome [13,14].

Periodontitis is a chronic inflammatory disease initiated by the colonization of subgingival periodontal pathogens. Although detecting the periodontal pathogens at the subgingival plaque should be the most definitive method to identify the cause of the disease, it requires skilled professionals for sample collection. It has been shown that periodontal microorganisms are not only restricted to subgingival pockets, but are also found on various mucous membranes in patients with periodontitis including buccal mucosa and supragingival plaque [15,16,17]. Thus, detecting periodontal pathogens in the buccal mucosa or supragingival space could be easily performed by non-professionals in a non-invasive manner, and aid the diagnosis of the disease. In addition, if a self-diagnostic device based on the peritonitis-associated bacterial panels is developed, it could be easily applied into the samples collected from the buccal mucosa or supragingival space.

A previous study showed that gut human metagenomic data from healthy subjects and patients can be used as biomarkers to predict diseases using machine learning algorithms [18]. Several studies also reported that selected features from the whole microbiome data and machine learning algorithms can be applied to predict the multiple diseases [19,20]. Interestingly, Chen et al. showed that the oral microbiome profiles can be potential biomarkers for arthritis screening [21], and the compositions of the microbiota communities from 76 subgingival plagues samples have been used to generate models for predicting the clinical status of patients with periodontal diseases [22]. However, the prediction of periodontitis using oral microbiome profiles from buccal mucosa or supragingival space has not been studied yet. Therefore, we hypothesized that the diversity of the microbiome community is different between the healthy group (control group) and the periodontitis group (disease group) in the buccal mucosa and supragingival space. In other words, we set a null hypothesis as: “There is no difference of microbiome diversity in healthy group and disease group.” To test the hypothesis, we characterized, compared, and tested the diversity of the oral microbiome profiles from the buccal mucosa and supragingival space, both in 62 healthy subjects and 210 periodontal patients in Korea. Furthermore, we clinically validated the identified periodontitis-associated microbiome, using the plaques from an independent cohort (105 healthy subjects and 116 periodontal patients) not used in the microbiome analysis. Additionally, we developed bioinformatic pipelines to identify and test potential microbial biomarkers for the early diagnosis of periodontitis and the monitoring of periodontitis progression.

## 2. Materials and Methods

### 2.1. Study Population and Clinical Examination

A total of 272 subjects (healthy subjects: 62, periodontitis subjects: 210) were recruited at the Department of Periodontics, Pusan National University Dental Hospital (Yangsan, Korea). Prior to clinical examination, full medical and dental history was obtained through interviews. In general, we excluded individuals who were pregnant or breastfeeding, had systemic diseases that may affect periodontal status, or had received antibiotics in the last 6 months or periodontal therapy (scaling and root planning) in the last 3 months. Similarly, a person on anti-inflammatory drugs, a subject with acute infection (e.g., herpetic gingivostomatitis) or chronic mucosal lesion (e.g., pemphigus, pemphigoid) of the oral cavity, or a smoker was excluded. Patients with periodontitis had moderate-to-severe periodontitis, including clinical attachment loss > 3 mm, and radiographic evidence of extensive bone loss. The healthy control group consisted of individuals with clinically healthy periodontal tissues (low scores of bleeding on probing in < 10% of the sites and no sites with PD > 3 mm or clinical attachment loss). Full-mouth clinical examinations including probing depth (PD), clinical attachment level (CAL), gingival index (GI), and plaque index (PI) were carried out by one practitioner. The experimental protocol was approved by the Institutional Review Board of Pusan National University. Even though the sample size in the current study was larger than those of previous microbiome studies associated with periodontal disease [14,23,24,25,26], a power analysis for sample size was performed using a web application of microbiome power calculator, which is based on Monte Carlo simulations and Wilcoxon–Mann–Whitney test [27]. The simulation results demonstrated that the current study with 272 samples had sufficient power (equal or greater than 0.8 for both buccal and supragingival parameters) to detect at least significant differences in community diversity between groups, as well as differences in abundance of the 20 most important operational taxonomic units (OTUs).

### 2.2. Plaque Sample Collection

Buccal and supragingival plaque samples were collected with the full-mouth periodontal examination. All participants were requested to refrain from food for 2 h and oral hygiene such as brushing or flossing the teeth for 2 h before sampling. Buccal swab samples were obtained from the mucosa of both cheeks. Supragingival plaque samples were collected from each participant from the mesiobuccal surface of upper or lower 1^st^ molars. Samples were collected after isolating the selected sampling site with cotton rolls and air drying gently. All samples were collected with a sterile microbrush and placed in a separate sterile 1.5-mL microcentrifuge tube. Plaque samples were stored at −80 °C for subsequent processing.

### 2.3. DNA Preparation and 16s rRNA Sequencing

DNA was extracted using MasterPure Gram positive DNA purification kit (Lucigen, Middleton, WI, USA) according to the recommended manufacture protocol. Each sequenced sample was used to prepare the sequencing library according to the Illumina 16S Metagenomic Sequencing Library protocols. The quality and quantity of the DNA sequencing library were assessed by PicoGreen (Molecular Probes, Eugene, OR, USA) and Nanodrop (Thermo Fisher Scientific, Waltham, MA, USA). The 16S rRNA genes were amplified from input genomic DNA using 16S V3-V4 primers and a subsequent limited-cycle amplification step was performed with multiplexing indices and Illumina sequencing adapters. The final products were pooled and normalized by the PicoGreen, and the size of libraries was verified using the LabChip GX HT DNA High Sensitivity Kit (PerkinElmer, Waltham, MA, USA). Finally, the libraries were sequenced using MiSeq or HiSeq 2500 platforms (Illumina, San Diego, CA, USA).

### 2.4. Microbiome Analysis and Statistical Analysis

Basic microbiome analyses have been performed using the QIIME2 [28] and associated plugins. To measure alpha diversities, abundance-based coverage estimator (ace) metric (for richness), Shannon’s index (for evenness), and Faith’s phylogenetic diversity (for alpha rarefaction plots) method were used. For the beta diversity analysis, the unweighted UniFrac distance was used. Using the distance matrix, principal coordinates analysis (PCoA) has been performed. Kruskal–Wallis pairwise tests and permanova tests were used to assess the statistical significances between or among groups for alpha and beta diversities, respectively. To assign taxonomy to the unique representative sequences, pre-trained Naive Bayes classifier, using Human Oral Microbiome Database (HOMD) 16S rRNA Extended RefSeq sequences (version 15.1) [29], were used. Centered log-ratio (CLR) method was used to compute the relative abundance of bacterial species with the average number of reads ≥ 5 in a sample, and the Pearson correlation was used to assess the association between bacterial species and demographic characteristics/clinical parameters. To statistically test differential abundance of bacterial species between healthy and periodontitis groups, DESeq2 [30] and LefSe [31] tools were used. For the prediction models for microbial biomarkers, five different machine learning algorithms, K nearest neighbor (KNN), LogitBoost, logistic model tree (LMT), support vector machine (SMO), and Naïve Bayes, implemented in WEKA 3.8 software [32] were used. The bacterial community interaction networks have been constructed using SparCC method [33] and visualized in Cytoscape software [34]. Detail bioinformatic methods were described in the Supplementary Methods.

### 2.5. Microbiome Validation using Real-Time Polymerase Chain Reaction (RT-PCR)

Species-specific primers and probes targeting the variable regions of the 16S ribosomal RNA (r RNA) of the selected strains were used for RT-PCR. Species-specific primers were designed by AlleleID (Premier Biosoft, Palo Alto, CA). List of primers used in this study is in Table S1. The reaction conditions for amplification of DNA were 95 °C for 10 min, 40 cycles of 95 °C for 30 s, and 60 °C for 30 s. The RT-PCR analyses were conducted using a real-time PCR system (Applied Biosystems, Thermo Fisher Scientific, Waltham, MA, USA). Total bacterial count was determined by 16S rRNA. The relative abundance of the target bacteria was calculated using the ∆∆Ct method and was normalized to 16S rRNA.

### 2.6. Data Avalialbity

The raw sequencing data have been deposited at NCBI GenBank under BioProject ID PRJNA576446 (BioSample SAMN12993997 - SAMN12994540).

### 2.7. Ethics Statement

All donors provided written informed consent, and their rights were protected according to the protocol that was reviewed and approved by the Institutional Review Board of Pusan National University (IRB No. PNUDH-2017-023).

## 3. Results

### 3.1. Microbial Community Diversities

In total, 272 subjects—62 healthy subjects (H) and 210 periodontitis subjects—were recruited and the plaque samples were obtained from both the buccal mucosa (B) and the supragingival site (S) (Table 1 and Table S2). The extracted and targeted amplified DNA (16S rRNA) was used for the sequencing library construction, followed by sequencing in the illumina platforms. The raw sequencing reads were processed using the QIIME2 pipelines [28]. A total of 42,090,259, quality-filtered sequencing reads were obtained at an average of 77,372 reads per sample and the filtered reads were grouped into 14,555 unique representative sequences (features). To perform accurate taxonomy assignments, we used a pretrained Naïve Bayes classifier using HOMD 16S rRNA Extended RefSeq sequences [29] and the features were successfully classified into 13 phyla, 28 classes, 50 orders, 93 families, 193 genera, and 527 species.

**Figure 1:**
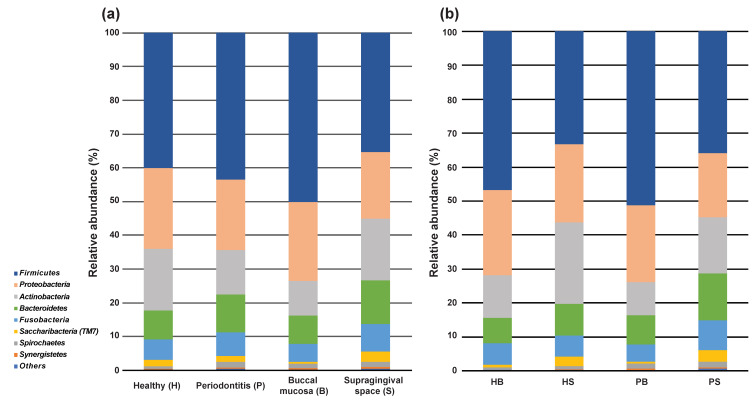
Relative abundance of bacterial phyla grouped by the disease status, sampling sites, and four subject groups. (**a**) Disease status; Healthy (H) vs. Periodontitis (P) and sampling sites; Buccal mucosa (**b**) vs. Supragingival space (S) (B) four subject groups; Healthy_Buccal (HB), Healthy_Supragingival (HS), Periodontitis_Buccal (PB), and Periodontitis_Supragingival (PS).

At the phylum level, the eight most abundant phyla were *Firmicutes*, *Proteobacteria*, *Actinobacteria*, *Bacteroidetes*, *Fusobacteria*, *Saccharibacteria* (*TM7*), *Spirochaetes*, and *Synergistetes*. Compared with the disease status subject groups (healthy and periodontitis), the relative abundance of *Firmicutes* and *Bacteroidetes* in the healthy group were slightly lower than in the periodontitis group (40.00% and 8.37% vs. 43.47% and 11.29%). On the other hand, *Proteobacteria* and *Actinobacteria* showed an opposite trend (24.05% and 18.29% vs. 20.86% 13.10%). In the case of the comparison between sampling sites, the relative abundance of *Firmicutes* was much higher in the buccal mucosa compared with the supragingival space (50.12% vs. 35.24%) (Figure 1a). We also divided the samples into the four subject groups (Healthy_Buccal: HB, Healthy_Supragingival: HS, Periodontitis_Buccal: PB, and Periodontitis_Supragingival: PS). The overall community composition in each subject group was depending on the sampling sites rather than the clinical status (Figure 1b).

**Figure 2:**
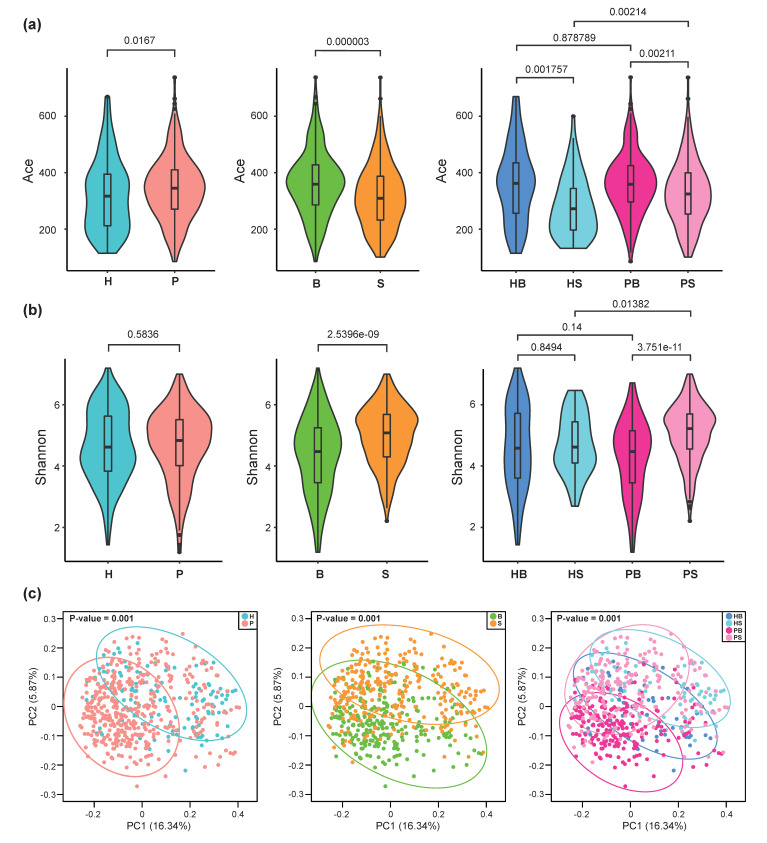
Alpha and beta diversities of bacterial communities grouped by the disease status, sampling sites, and four subject groups. Kruskal–Wallis pairwise test was used to assess the statistical significance between groups for the alpha diversities. In case of beta diversities, permanova test was used to evaluate the statistical significance between groups. (**a**) Alpha diversity—richness (abundance-based coverage estimator; Ace) (**b**) alpha diversity—evenness (Shannon diversity index), (**c**) Principal coordinate analysis plots based on unweighted UniFrac distance. Adjusted P values provided on each plot are for the indicated comparisons.

The richness of the total amount of bacteria measured by Ace index and the rarefaction curves of community diversity (faith’s phylogenetic diversity) for the disease status and sampling sites showed that the diversity in the periodontitis group and the B group was higher (Figure 2a and Figure S1), although the evenness (Shannon index) between the H group and periodontitis groups was not significantly different (Figure 2b). Similar to the relative abundance result, the alpha diversity among four subject groups was influenced by the sampling site (right panels of Figure 2a,b). The beta diversity analysis revealed that the differences for all comparisons in disease status, sampling site, and four subject groups were statistically significant. PCoA analysis showed that the individual samples were clustered in separate groups (Figure 2c).

### 3.2. Identification of Bacterial Species Relevant to Clinical Parameters and Enriched in the Healthy and Periodontitis Groups

**Figure 3:**
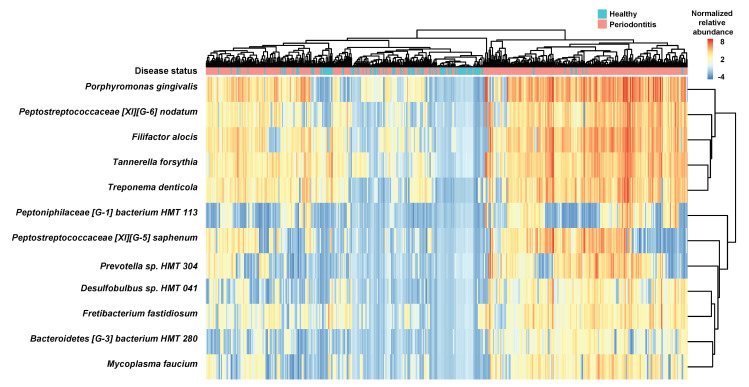
A heatmap of 12 highly correlated bacterial species. Pearson correlation was used to evaluate the association between bacterial abundances and clinical parameters (age, gender, PD, CAL, GI, and PI) and if the adjusted p-value was less than 0.001, the bacterial species was defined as a highly correlated bacterial species (Table S3). The heatmap was generated using the relative abundance normalized by centered log-ratio (CLR) methods (see supplementary methods).

To identify the bacterial species associated with the demographic characteristics, age and gender, and four clinical parameters, correlations between the normalized bacterial relative abundance and the measurements of parameters were calculated using the Pearson correlation method. A total of 12 species were significantly associated with 5 parameters (Age, PD, CAL, GI, and PI) (Table S3). Interestingly, *P. gingivalis*, a well-known specific periodontal pathogen, was associated with all parameters except gender and the other specific periodontal pathogens, *T. denticola* and *T. forsythia* were associated with PD, CAL, and GI. A heatmap plot for the distributions of 12 highly associated species clearly showed that the relative abundance profiles of bacterial species in periodontitis group were pretty similar (Figure 3).

**Figure 4:**
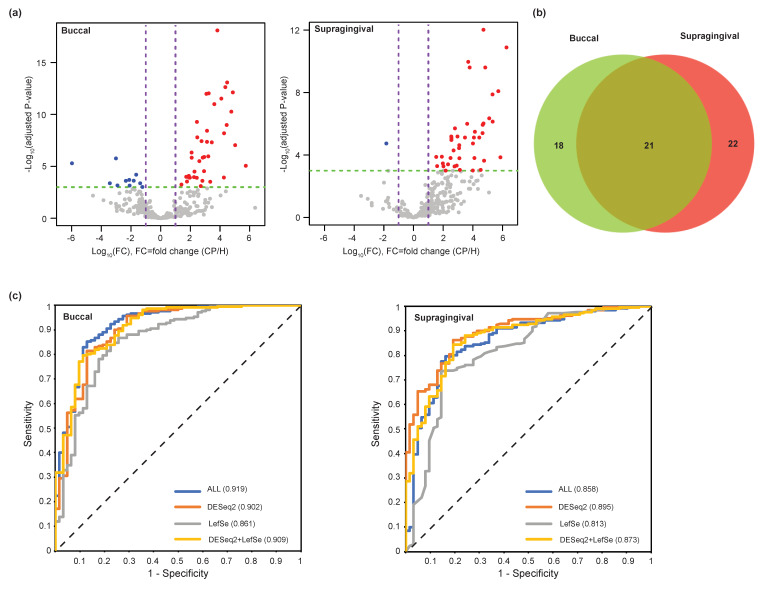
Bacterial species enriched in the H and periodontitis groups. (**a**) Volcano plots for the bacterial species for the different bacteria species abundance. DESeq2 was to use to test the statistical significance. Differential abundance of bacteria species was defined as those with changes of at least 4-fold between samples at a false discovery rate (FDR) of 0.001%. The x and y axes in the plot present the magnitude of fold changes (log2 transformed) and the adjusted p-value (-log10) by Benjamini–Hochberg correction, respectively. Dominant bacterial species in the P group were represented as red dots and dominant bacterial species in the H group were shown as blue dots in the plot. (**b**) Venn diagram of the number of differential bacterial species common/unique to the buccal (green) and supragingival (red) sites. (**c**) The ROC plots and AUC for four different feature sets. ALL; all bacteria species, DESeq1; the enriched bacterial species identified DESeq2, LefSe; the enriched bacterial species identified LefSe, and DESeq2+LefSe; union of DESeq2 and LefSe sets. Right panel: buccal site samples with logistic model tree (LMT) algorithm and left panel; supragingival site samples with LogitBoost algorithm.

Next, we investigated the bacterial species that demonstrated significant differences in their abundances between the H and periodontitis groups. When we did the test using DESeq2 method [30], 50 (buccal site, H: 11 and P: 39) and 43 species (supragingival site, H:1 and P: 43) were differentially distributed with statistical significance between two groups (Figure 4a and Table S4). When we checked the bacterial species enriched in the periodontitis between two sampling sites, 21 bacterial species were common to both sampling sites (Figure 4b) and the overlap between two groups was statistically significant (p-value: 1.607×10^−15^, hypergeometric test). We also performed a similar analysis using the LefSe method [31] (Figure S2). Through both analyses, we found 8 species in both buccal and supragingival samples of periodontitis patients significantly increased compared with the healthy subjects. The “red” complex including *P.gingivalis*, *T. forsythia*, and *T. denticola* were significantly increased in both buccal and supragingival samples in periodontitis patients. In addition, *F. alocis*, *P. endodontalis*, *F. fastiosum*, and *Peptostreptococcus* species were also significantly increased in both sampling sites in periodontitis patients. In buccal samples, *M. faucium*, *C. rectus*, and several *Prevotealla* species were increased as well. In supragingival samples, *Treponema* species including *T. socranskii* and *Prevotella* species were also significantly increased. Taken together, many of the periodontal pathogens that have been reported to be found in the subgingival plaques were also detected in buccal and supragingival samples and significantly increased in periodontitis patients. These results indicate that bacterial composition of subgingival plaque associated with periodontitis can be predicted by microbiome analysis of buccal mucosa or supragingival plaque samples without analyzing subgingival plaque microbiome.

### 3.3. Evaluation of Microbial Biomarkers for the Periodontitis

To discover the potential microbial biomarkers for periodontitis, we established a pipeline to test and evaluate the prediction models. In total, 266 and 259 filtered bacterial species were used for the input feature dataset to generate prediction models in buccal and supragingival sample sites, respectively. First, we evaluated the performance of the classification using all bacterial species (ALL) and five different machine learning algorithms including KNN, LMT, LogitBoost, SMO, and Naïve Bayes (Table S5). The highest accuracy for the buccal site was 89.3% (LMT algorithm) and for the supragingival site, it was 83.8% (LogitBoost). The F_1_ score performance measurement for the tests was between 0.910 and 0.931 (buccal) and 0.805–0.899 (supragingival). The sensitivity for the tests was between 0.876 and 0.967 (buccal) and 0.729–0.952 (supragingival), and the specificity was between 0.468 and 0.839 (buccal) and 0.306–0.726 (supragingival). Next, we explored subsets of bacterial species to establish potential core microbial biomarkers. We used the identified bacterial species having differential abundance between the H and periodontitis groups. Three different feature subsets: (1) the enriched bacterial species identified DESeq2 (DESeq2), (2) the enriched bacterial species identified LefSe (LefSe), and (3) the union of the DESeq2 and LefSe sets (DESeq2+LefSe) were used to perform the same classification tasks done by the ALL set. In the case of the buccal site, the best performance was achieved by using the DESeq2+LefSe subset and KNN algorithm, and the accuracy and F_1_ score for the test were comparable to the use of all bacterial species (For accuracy, ALL: 89.3% vs. DESeq2+LefSe: 88.6%; For F_1_ score, ALL: 0.931 vs. DESeq2+LefSe: 0.926). In the case of the supragingival site, the DESeq2+LefSe subset and LogitBoost algorithm gave the best performance and more interestingly, the accuracy and F_1_ score of the test were higher than the ones of the test with the ALL set (For accuracy, ALL: 83.8% vs. DESeq2+LefSe: 85.3%. For F_1_ score, ALL: 0.899 vs. DESeq2+LefSe: 0.905). Additionally, we used the area under the ROC curve (AUC) to evaluate the performance of the prediction models generated by different feature subsets and algorithms (Table S5). The results demonstrated that the performance using the DESeq2 or DESeq2+LefSe subsets were similar or comparable to the ALL set in the buccal site (Figure 4c). For the supragingival site, the AUC value of DESeq2 subset case was the highest among all tests (Figure 4c). Therefore, the current analysis revealed that oral microbiome profiles were able to distinguish the healthy subjects and periodontitis patients and that the selected bacterial species could be used as potential microbial biomarkers to diagnose periodontitis.

### 3.4. Clinical Valdiation of the Identified Periodontitis-Associated Microbiome

**Figure 5:**
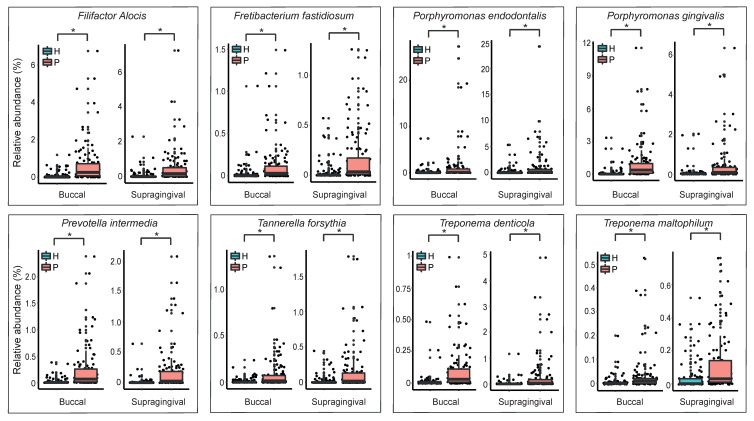
Clinical validation of the identified microbiome related to periodontitis. Real-time PCR (RT-PCR) was performed to measure a relative abundance of bacterial species in the sample. The difference between two groups (H: healthy group, P: periodontitis group) was assessed using the Wilcoxon rank sum test. The * indicates that the p-value for the test is less than 0.01.

To clinically evaluate the periodontitis related microbiome identified from the microbiome analysis, we selected 8 bacterial species (*Filifactor alocis, Fretibacterium fasdtidiosum, Porphyromonas endodontalis, Porphyromonas gingivalis, Prevotella intermedia, Tannerella forsythia, Treponema denticola,* and *Treponema maltophilum*) enriched in the periodontitis group from both the buccal mucosa and supragingival space and measured the relative abundance of those bacterial species in the subjects from an independent cohort (total of 221 individuals; 105 healthy subject and 116 periodontal patients) which was not used for the analysis. The same sampling procedure has been performed to obtain plaques from the buccal mucosa and supragingival area from the subject in the cohort. RT-PCR analysis of the 8 bacterial species showed that the relative abundance of the healthy group and the periodontitis group was significantly different (Figure 5).

### 3.5. Bacterial Community Interaction Networks

**Figure 6:**
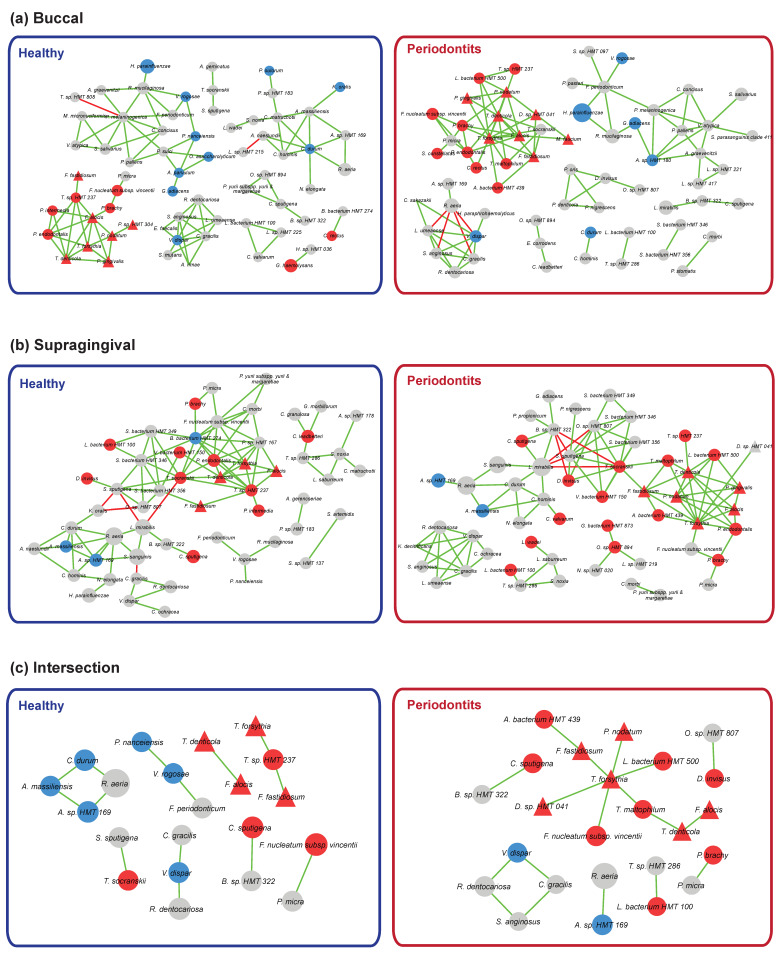
Bacterial community interaction network analysis. The co-occurrence bacterial community networks were constructed using SparCC method and visualized in Cytoscape software. The intersection networks between two networks between buccal and supragingival sites were obtained using Cytoscape merge tools. (**a**) Interaction networks in buccal site, (**b**) interaction networks in supragingival site, and (**c**) intersection networks in the H and periodontitis groups. Diamond shape node represents the highly correlated bacterial species with clinical parameters. The differential bacterial species between healthy and periodontitis groups were colored as blue (healthy) or red (periodontitis). The directions of the correlation between two bacterial species were annotated as edge colors; red color: negative correlation, green color: positive correlation.

Previously, it has been reported that oral dysbiosis characterized by an imbalance in the relative abundance of microbial species and a shift from beneficial symbiotic bacteria to pathogenic bacteria play important roles in the development of periodontal diseases [6]. We investigated the oral dysbiosis by checking the interactions among bacterial species in healthy and periodontitis groups. First, we assessed the co-occurrence of the bacterial species by constructing correlation networks. The top 100 significant absolute correlations among bacterial species were selected and visualized in Figure 6a,b. In the case of the buccal site, several independent modules existed and among them, a distinct module consisting of periodontal pathogens (red nodes) such as *Porphyromonas*, *Treponema*, *Tannerella*, *Filifactor*, and *Prevotella* was identified in both healthy and periodontitis groups. Interestingly, the size of the module (the number of species node) in the periodontitis group was larger than the one in the healthy group. When we checked the average number of neighbors which is indicating the average connectivity of a node in the network, the average number of neighbors in the periodontitis network (3.125) was greater than the one in the healthy network (3.030). Therefore, the connectivity of the module in the periodontitis group was denser than the one in the healthy group. On the other hand, in the case of the supragingival site, there was a big network module including both commensal bacteria species and periodontal pathogens. Similar to the buccal site, more periodontal pathogens and higher connectivity were observed in the periodontitis group (the average number of neighbors; periodontitis: 3.636 vs. healthy: 3.571). Next, we extracted common modular structures between the networks in healthy and periodontitis groups from two sampling sites. As expected, we identified a noticeable interconnected module consisting of 10 periodontal pathogens in the periodontitis group. In the case of the healthy group, we observed few small sub-modules including the bacterial species enriched in the healthy group identified (Figure 6c). Overall, the network-based approach analysis has shown that even though commensal bacteria and periodontopathogens were preserved in the network for both healthy and periodontitis groups, the network topology of the interconnected module consisting of periodontopathogens was altered and furthermore, the connectivity of the module was enforced.

## 4. Discussion

Periodontitis is a chronic inflammatory disease caused by colonization of subgingival periodontal pathogens. It has been shown that periodontal microorganisms are not only restricted to subgingival pockets, but are also found on various mucous membranes in patients with periodontitis [14,16,17]. Microbiota on buccal mucosa and in supragingival area can be collected easily, noninvasively, and repetitively. Therefore, sampling the microbiota on oral mucosa or supragingival area is a promising way to diagnose periodontal diseases. Some investigations have demonstrated that the buccal mucosa microbiota in periodontally healthy and periodontitis subjects differ, but were almost based on traditional identification of bacteria in cultivations or culture-independent molecular studies [35]. Many studies using NGS have used subgingival plaque to assess the composition of the microbial periodontal community [24,25,36]. The subgingival plaque sampling has an advantage in more accurately monitoring the condition of periodontitis than the sampling of other sites. However, the subgingival plaque sampling is an invasive method and it requires specialized techniques to perform. In addition, if not properly sampled, bacteria species from nearby the subgingival sites may be contaminated, and the results of the bacterial community analysis could be in the wrong direction. Therefore, there is a need for a sampling site that is easy to carry out in a non-invasive manner and that reflects the condition of periodontitis. To identify reliable alternative sampling sites, it is important to obtain and analyze a large number of samples. In the current study, we collected a total 544 plaques samples (both buccal mucosa and supragingival site) from 272 subjects (62 healthy subjects and 210 patients with periodontitis) to increase the reliability of the analysis. Up to this date, only few studies have reported that buccal mucosal membrane and supragingival sampling could serve as alternative sources for detecting and enumerating known and novel bacterial biomarkers of periodontitis [14,15]. Thus, one of purpose of this study was to determine if buccal and supragingival sampling could be used as a non-invasive detection method of indicator taxa for periodontitis instead of conventional subgingival plaque sampling. A relatively simpler alternative sampling protocol would greatly facilitate the detection of periodontitis-associated bacterial biomarkers in disease monitoring.

In our research, we used 16S rRNA gene sequencing to evaluate and compare the different characteristics of buccal and supragingival samples between healthy subjects and periodontitis patients. The richness of the total amount of bacteria measured by alpha diversity showed that the diversity in the periodontitis group was higher. Other previous studies have also reported that the bacterial diversity was higher in periodontitis patients compared with the healthy subjects [14,24]. We hypothesized that the microbiome community diversity is different between the healthy group (control group) and the periodontitis group (disease group) in the buccal mucosa and supragingival space in the present study. The community diversity analyses (alpha and beta diversities) showed that there is a statistically significant difference between the healthy group and the periodontitis group. Therefore, we were able to reject the null hypothesis, “There is no difference of microbiome diversity in healthy group and disease group”, which means that there was difference of the bacterial composition between the healthy group and the periodontitis group. To identify the bacterial species associated with the demographic characteristics (age and gender) and four clinical parameters (PD, CAL, GI, and PI), correlations between bacterial relative abundance and the measurements of parameters were calculated. A total of 12 species were significantly associated with 5 parameters (Age, PD, CAL, GI, and PI). Interestingly, *P. gingivalis* was associated with all parameters except gender, and *T. denticola* and *T. forsythia* were associated with PD, CAL, and GI. Chen et al. have reported that subgingival *P. gingivalis*, *T. denticola*, and *T. forsythia* exhibit a very strong relationship with pocket depth and clinical attachment level [22]. Also, Texal et al. have reported that at low supragingival plaque levels, *P. gingivalis* was significantly associated with CAL, while at high supragingival plaque levels, *T. forsythia* and *P. gingivalis* was associated with CAL [37].

Next, we investigated the bacterial species that demonstrated significant differences in their abundances between periodontitis patients and healthy subjects. Through DESeq2 and LefSE analysis, we found 8 species in both buccal and supragingival samples of periodontitis patients that were significantly increased compared with the healthy subjects. Among them, the “red” complex including *P. gingivalis*, *T. forsythia*, and *T. denticolar* was significantly increased in both buccal and supragingival samples in periodontitis patients. In addition, *F. alocis*, *P. endodontalis*, *Fretibacterium fastiosum*, and *Peptostreptococcus* species were also significantly increased in both sampling sites in periodontitis patients. *F. alocis* has been identified as a potential pathogen in previous studies [38,39]. It is relatively resistant to oxidative stress and can induce inflammatory responses. Moreover, in co-culture with *P. gingivalis*, *F. alocis* showed increased invasiveness [40]. *P. endodontalis* has been reported to be associated with periodontitis [12]. Although *P. endodontalis* is highly sensitive to oxygen and is difficult to cultivate from clinical samples [17], it was detected in both buccal and supragingival samples in periodontitis patients. Peptostreptococcus is also known as a potential periodontal pathogen and it can coaggregate with *Fusobacterium nucleatum* and *P. gingivalis* [41]. *Fretibacterium* belongs to the phylum *Synergistetes* and has been detected in subgingival plaque from periodontitis patients [42,43]. Taken together, our result suggests that periodontal pathogens increase not only in the subgingival space, but also in the buccal mucosa and supragingival space. Thus, their presence can be detected in these sampling sites to aid the diagnosis of periodontitis. More importantly, we clinically validated the periodontitis-associated microbiome using an independent cohort which consisted of 211 individuals.

It is well known that the dysbiosis characterized by an imbalance of microbial species abundance plays an important role for the transition from periodontal health to periodontitis [6]. As expected, we observed that periodontal pathogens were highly enriched in the periodontitis group, which could be a signature of a shift from beneficial symbiotic bacteria to pathogenic bacteria. However, it is still difficult to answer the question of how overall relationships among bacterial species would be altered in periodontitis just by inspecting the increase of periodontal pathogens in the periodontitis group. To address the question, we constructed the co-occurrence network and identified a distinctive module consisting of known periodontopathogens that were observed in both H and periodontitis groups. Remarkably, the module had many positive co-correlations among periodontopathogens in the periodontitis group, and the conserved network structure patterns between buccal and supragingival sites were detected. These results fairly agreed with the previous oral microbiome studies [23,26]. Thus, we reveled that it is important to consider not only the abundance of periodontopathogens, but also the relationships of colonized bacterial species to understand how oral pathogens boost the pathogenicity of a polymicrobial community in periodontitis.

In the present study, we used four different sets of oral microbiomes from buccal and supragingival mucosa to generate the prediction models for periodontitis. The performances of five machine learning approaches were evaluated with these sets. The ALL set with LMT and the DESeq2+LefSe set with LogitBoost showed the best performance in the buccal and supragingival site, respectively. By evaluating the performance of the prediction models, we showed that oral microbiome abundance can be used as an effective measurement to predict periodontitis. Moreover, the optimal subsets of oral microbiome abundance in buccal mucosa and supragingival plaque can be potential microbial markers for the diagnosis of periodontitis. These results suggest that buccal and supragingival sampling could be used as a non-invasive method of a diagnosis of periodontitis, instead of subgingival plaque sampling. In conclusion, we have identified species that were significantly increased in buccal and supragingival samples from periodontitis patients and further evaluated their possibility in detecting periodontitis through machine learning. 

This is the first report to show the possibility to diagnose periodontitis even at an early stage or monitoring the progress of periodontitis by detecting specific periodontal pathogens in the buccal mucosa or supragingival plaque. From a clinical standpoint, this study provides an important groundwork for developing a minimally invasive and convenient method to monitor periodontitis.

## Figures and Tables

**Table 1:** Demographic characteristics and clinical parameters of healthy subjects and patients with periodontitis (mean ± standard deviation).

Characteristic	Healthy (n = 62)	Periodontitis (n = 210)	P-Value
Age (years)	28.45 ± 8.64	54.28 ± 10.92	< 0.01
Gender	^1^M: 25, ^2^F: 37	M: 116, F:94	^3^NS
Full mouth examination			
^4^PD (mm)	2.4 ± 0.21	3.59 ± 0.92	< 0.01
^5^CAL (mm)	2.41 ± 0.21	4.08 ± 1.13	< 0.01
^6^GI	0.12 ± 0.11	0.98 ± 0.52	< 0.01
^7^PI	16.48 ± 14.17	61.02 ± 24.17	< 0.01

^1^M: Male, ^2^F: Female, ^3^NS: not significant, ^4^PD: probing depth, ^5^CAL: clinical attachment level, ^6^GI: gingival index, ^7^PI: plaque; For the Age, PD, CAL, GI, and PI, the difference between two groups was assessed using the Wilcoxon rank sum test. For the Gender, Pearson’s Chi-squared test with Yates’ continuity correction was used.

## Supplementary Materials

The following are available online at https://www.mdpi.com/2077-0383/9/5/1549/s1, Figure S1: Rarefaction curves of community diversity (faith’s phylogenetic diversity) for the disease status; healthy (H) and periodontitis (P), sample sites; buccal mucosa (B) and supragingival space (S) and the four subject groups; HB, HS, PB and PS., Figure S2: Differential abundance analysis of bacterial species by LefSe method, Table S1: A list of primers used for real-time PCR analysis. Table S2: The demographic characteristics and clinical parameters of subjects, Table S3: Summary of the correlation analysis of bacterial species with demographic characteristics and clinical parameters. Table S4: Differential abundance of bacterial species using DESeq2 method, Table S5: Evaluation of prediction models using four feature sets and five machine learning algorithms.
