# Supplementary material for: Identification of Potential Oral Microbial Biomarkers for the Diagnosis of Periodontitis
Source: J Clin Med. 2020 May 20;9(5):1549. doi: 10.3390/jcm9051549 (PMC7290295; doi:10.3390/jcm9051549)
Supplement: Supplementary file 1 [file jcm-09-01549-s001.pdf]

Supplementary Methods

16S rRNA (ribosomal RNA) sequencing metagenomic data processing

The adapter sequences of raw sequencing reads were trimmed using cutadapt tool (https://cutadapt.readthedocs.io/en/stable/) and the trimmed sequencing reads were filtered using Deblur method [1] implemented in QIIME2 pipelines (2019.4 version) [2]. In this step, the individual reads in each library were further trimmed based on the raw read quality profiles and each sequencing read was grouped into unique representative sequences called as features (previously represented as OTUs). To perform "Faith's Phylogenetic Diversity" and "unweighted UniFrac analysis", the phylogenetic tree was constructed using QIIME2 plugin, "qiime phylogeny align-to-tree-mafft-fasttree".

Diversity analyses

Alpha and beta diversity analyses have been performed using QIIME2 plugins, "qiime diversity alpha" and "qiime diversity beta" respectively. For the alpha diversity analysis, two different metrics were used. For the richness measurement, "Abundance-based Coverage Estimator (ACE) metric" was calculated and "Shannon's index" was calculated for the evenness measurement. To perform alpha rarefaction plotting, "qiime diversity alpha-rarefaction" plugin was used, and the rarefaction plots were visualized using "Faith's Phylogenetic Diversity" measurement. To test for the associations between categorical metadata (disease status, sampling sites and four subject groups), Kruskal-Wallis pairwise tests were performed.

For the beta diversity analysis, "unweighted UniFrac" method was used to calculate the distance between the features. Using the constructed distance matrix, Principal Coordinate Analysis (PCoA) which reduce which extracts features and reduce dimensionality of the data, has been performed and the first two principal coordinates were used to plot the individual samples. To analyze sample composition in the context of categorical metadata, we used permanova tests.

Assigning bacterial taxonomy to the features

The assignment process has been performed using Naïve Bayes classifier implemented in "qiime feature-classifier" QIIME2 plugin. First, the classifier was constructed using Human Oral Microbiome Database (HOMD) 16S rRNA Extended RefSeq sequences (version 15.1) [3] and then, the individual feature sequences were classified into the different level of taxonomy using the pre-trained Naïve Bayes classifier.

Bacterial species correlation to the demographic characteristics/clinical parameters

To assess the associations between bacterial species and demographic characteristics/clinical parameters, the relative abundance of bacterial species with the average number of reads $\geq$ 5 in a sample was calculated by centered log-ratio (CLR) method. Pearson correlation was computed and if the p-value for the correlation is less than 0.001, we defined that the bacterial species was highly associated with characteristics or parameters.

Differential abundance of bacteria species between healthy and periodontitis groups

To identify the bacteria species distributed differently between healthy and periodontitis groups, DESeq2 [4] and LefSe [5] tools were used. In case of DESeq2, the adjusted p-value is less than or equal to 0.001 and the absolute fold-change is greater than or equal to 4, we defined the differential abundance of bacteria species as significant. For the LefSe method, we used the default threshold (2) on the logarithmic LDA score for discriminative features.

Evaluation of the prediction models

To predict the periodontal disease status using microbiome profiles, we used the normalized relative abundance described in the above section. Five different machine learning algorithms; K nearest neighbor (KNN), LogitBoost, logistic model tree (LMT), support vector machine (SMO), Naïve Bayes implemented in WEKA 3.8 software [6] have been used for the prediction and evaluation for prediction models. Four different bacterial species sets; ALL; all bacteria species, DESeq1; the enriched bacterial species identified DESeq2, LefSe; the enriched bacterial species identified LefSe and DESeq2+LefSe; union of DESeq2 and LefSe sets, were used as input features for the classification tasks. To evaluate of the prediction models, 10-fold cross-validation approach was used. For the performance assessment, accuracy and the area under ROC curve (AUC) were used. In case of KNN, SMO and LogitBoost algorithms, we explored and chose the parameters to give us the best results (highest accuracy). For the KNN, the parameter KNN was explored from (1...20). For the SMO, RBFKernel was used and the parameter Gamma and cacheSize were selected in (0.00001, 0.0001, 0.001, 0.01, 0.1, 1, 10, 100, 1000) and (0.01, 0.1, 1, 10, 100, 200, 300, 400, 500, 1000, 2000, 5000) respectively. For the LogitBoost, the parameter numIterations was chosen in (1…50).

Bacterial species interaction network analysis

To construct the co-occurrence networks, we used the SparCC methods [7] and the raw read counts for the bacterial species with the average relative abundance $\geq 0.05\%$. Default SparCC parameters have been used and to estimate the pseudo two-sided p-values 1,000 bootstraps have been performed. If the p-value for the correlation between bacterial species was less than 0.001, the correlation was considered as significant. The 100 highest absolute correlations were selected and used to construct a correlation network. The networks were visualized using Cytoscape software [8] and the intersection networks between buccal and supragingival site networks were obtained by "merge" tool implemented in Cytoscape.

References

1. Amir, A.; McDonald, D.; Navas-Molina, J.A.; Kopylova, E.; Morton, J.T.; Xu, Z.Z.; Kightley, E.P.; Thompson, L.R.; Hyde, E.R.; Gonzalez, A., et al. Deblur Rapidly Resolves Single-Nucleotide Community Sequence Patterns. *mSystems* **2017**, *2*, 7, doi:10.1128/mSystems.00191-16.
2. Bolyen, E.; Rideout, J.R.; Dillon, M.R.; Bokulich, N.A.; Abnet, C.C.; Al-Ghalith, G.A.; Alexander, H.; Alm, E.J.; Arumugam, M.; Asnicar, F., et al. Reproducible, interactive, scalable and extensible microbiome data science using QIIME 2. *Nat. Biotechnol.* **2019**, *37*, 1091-1091, doi:10.1038/s41587-019-0252-6.
3. Chen, T.; Yu, W.H.; Izard, J.; Baranova, O.V.; Lakshmanan, A.; Dewhirst, F.E. The Human Oral Microbiome Database: a web accessible resource for investigating oral microbe taxonomic and genomic information. *Database* **2010**, 10.1093/database/baq013, 10, doi:10.1093/database/baq013.
4. Love, M.I.; Huber, W.; Anders, S. Moderated estimation of fold change and dispersion for RNA-seq data with DESeq2. *Genome Biol.* **2014**, *15*, 38, doi:10.1186/s13059-014-0550-8.
5. Segata, N.; Izard, J.; Waldron, L.; Gevers, D.; Miropolsky, L.; Garrett, W.S.; Huttenhower, C. Metagenomic biomarker discovery and explanation. *Genome Biol.* **2011**, *12*, 18, doi:10.1186/gb-2011-12-6-r60.
6. Frank, B.; Hall, M.A.; Witten, I.H. *The WEKA Workbench. Online Appendix for "Data Mining: Practical Machine Learning Tools and Techniques"*, Fourth ed.; Morgan Kaufmann: 2016.
7. Friedman, J.; Alm, E.J. Inferring Correlation Networks from Genomic Survey Data. *PLoS Comput. Biol.* **2012**, *8*, 11, doi:10.1371/journal.pcbi.1002687.
8. Otasek, D.; Morris, J.H.; Boucas, J.; Pico, A.R.; Demchak, B. Cytoscape Automation: empowering workflow-based network analysis. *Genome Biol.* **2019**, *20*, doi:10.1186/s13059-019-1758-4.

Table S1. A list of primers used for real-time PCR analysis.

Species	Forward primer	Reverse primer
16S universal	CCTACGGGAGGCAGCAG	ATTACCGCGGCTGCTGGCA
Filifactor alocis	GCGAAGAACCTTACCTAAAC	CCACCTGTATCCATTGTCTA
Fretibacterium fastidiosum	CAGGCTGTGATTCAAGTC	ACCGATGTTCTTCCCAATA
Porphyromonas endodontalis	ATGATGGCAGATGAGAGTT	ATAGAGTCCTCAGCATAACC
Porphyromonas gingivalis	CGTAGGTTGTTCGGTAAGT	AGTGTCAGTCGCAGTATG
Prevotella intermedia	GACGCAGGATACAGAGAT	GATGGCAACTAAGGAAAGG
Tannerella forsythia	ATAGATGAAGTAGGCGGAAT	TCAGTGTCAGTTATACCTTAGT
Treponema denticola	AAGCCTGGTGTGAAATCT	CGTATATCAGCGTCAATCATC
Treponema maltophilum	TGAGACAGCATTGAGAACT	CCTTCGCCATTGGTATTC

Table S2. The demographic characteristics and clinical parameters of subjects.

Subject	Disease status	Age	Gender	PD	CAL	GI	PI
1	Periodontitis	56	Male	4.19	4.327	0.92	25
2	Periodontitis	41	Female	2.321	2.5	1.15	46.42
3	Periodontitis	51	Female	3.039	3.126	0.42	46.49
4	Periodontitis	34	Male	4.32	4.734	1.62	100
5	Periodontitis	35	Male	5.527	5.527	2	68.33
6	Periodontitis	61	Female	3.529	3.529	0.54	15
7	Periodontitis	38	Male	5.873	6.408	2.05	93.1
8	Periodontitis	52	Female	3.259	3.641	0.11	41.96
9	Periodontitis	43	Male	3.84	4.463	1.45	75
10	Periodontitis	60	Female	2.964	2.964	0.95	54.46
11	Periodontitis	63	Male	2.148	2.148	0.14	29.69
12	Periodontitis	57	Male	3.347	3.347	1.29	53.12
13	Periodontitis	53	Male	4.82	5.403	1.4	42.59
14	Periodontitis	60	Female	3.113	3.173	1.14	54.46
18	Periodontitis	59	Male	2.934	2.994	1.16	54.46
19	Periodontitis	64	Male	3.043	3.376	0.2	47.58
20	Periodontitis	56	Female	3.444	3.543	0.94	56.48
22	Periodontitis	40	Male	3.794	4.467	0.63	41.07
23	Periodontitis	55	Female	3.886	3.993	1.57	68.52
24	Periodontitis	69	Male	2.666	3.679	0.24	52.59
25	Periodontitis	64	Female	2.737	3.685	0.37	35.18
26	Periodontitis	55	Female	3.059	3.089	0.67	48.21
27	Periodontitis	45	Male	4.152	4.35	1.54	26.61
28	Periodontitis	58	Female	3.208	3.696	0.65	65.17
29	Healthy	28	Male	2.517	2.517	0.16	83.92
30	Healthy	64	Female	1.614	1.614	0.04	0.96
31	Healthy	54	Female	2.128	2.128	0.04	8.65
32	Healthy	28	Female	1.733	1.733	0.02	0
33	Healthy	37	Female	2.511	2.511	0.05	8.03
34	Periodontitis	69	Female	2.821	2.821	0.86	71.42
35	Healthy	53	Female	1.944	2.022	0	15.32
36	Healthy	23	Male	1.961	1.961	0.03	7.5
37	Healthy	26	Male	2.446	2.446	0.07	6.25
38	Periodontitis	60	Female	4.91	4.982	2	26.78
39	Periodontitis	53	Male	3.037	3.104	0.47	26.72
40	Periodontitis	54	Male	3.641	3.75	0.79	38
41	Periodontitis	47	Female	4.666	4.666	0.842	52.63
42	Periodontitis	61	Male	3.172	3.607	0.83	53.91
44	Periodontitis	53	Male	3.489	3.51	1.61	100
45	Periodontitis	45	Female	3.811	4.492	0.64	38.54
46	Periodontitis	68	Female	1.933	2.92	0.98	67
47	Periodontitis	49	Male	3.487	4.296	1.39	61.61
48	Periodontitis	67	Female	2.625	3.648	0.59	75.89

49	Periodontitis	73	Female	2.486	3.923	0.844	77.08
50	Periodontitis	49	Male	2.222	2.672	0.25	37.5
51	Periodontitis	53	Male	4.928	4.928	1.26	73.81
52	Periodontitis	58	Male	2.666	2.747	0.81	50
54	Periodontitis	50	Female	4.898	5.142	0.22	21.43
55	Periodontitis	53	Male	4.006	4.487	1.17	100
56	Periodontitis	57	Male	3.083	3.285	0.54	46
57	Periodontitis	52	Male	2.432	2.5	0.15	98.15
58	Periodontitis	55	Male	4.75	7.407	1.42	31.94
59	Periodontitis	57	Female	3.92	3.92	0.69	48.15
60	Periodontitis	67	Male	2.685	2.685	1.03	39.06
61	Periodontitis	54	Male	2.113	2.934	0.29	63.39
62	Periodontitis	45	Male	4.97	4.97	0.64	76.14
63	Periodontitis	49	Male	3.41	3.696	0.61	54.46
64	Periodontitis	53	Female	3.119	3.583	1.34	18.75
65	Periodontitis	72	Male	2.54	2.73	0.46	51.85
66	Periodontitis	52	Female	1.869	2.41	0.16	25
67	Periodontitis	52	Female	3.029	3.053	1.23	56.25
68	Periodontitis	39	Male	4.14	4.83	1.36	26
69	Periodontitis	62	Female	3.231	3.971	1.01	35.16
70	Periodontitis	40	Male	3.615	3.685	1.2	87.5
71	Periodontitis	50	Female	2.76	2.86	1.34	30.83
72	Periodontitis	40	Female	3.135	3.135	0.7	38.28
73	Periodontitis	61	Male	5.013	5.74	1.02	50
74	Periodontitis	54	Male	4.88	5.37	1.74	96.77
75	Periodontitis	48	Female	4.067	4.067	0.36	42.19
76	Periodontitis	62	Male	4.9	5.333	1.71	78
77	Periodontitis	65	Male	3.173	3.275	0.13	47.12
78	Periodontitis	46	Male	4.63	4.63	0.93	21.43
79	Periodontitis	59	Female	3.833	3.851	1.6	77.68
80	Periodontitis	61	Female	3.86	5.246	1.59	58
81	Periodontitis	73	Female	2.72	2.72	0.55	48.91
82	Periodontitis	61	Male	3.3	3.706	1.56	100
84	Periodontitis	45	Male	3.875	3.97	1.24	64.28
85	Periodontitis	37	Female	3.41	3.54	0.2	4.032
86	Periodontitis	48	Male	2.904	3.047	0.45	35.71
87	Periodontitis	77	Male	2.23	2.84	0.81	61.54
88	Periodontitis	46	Male	4.25	4.88	0.71	53.57
89	Periodontitis	74	Female	3.384	3.683	0.86	51.85
90	Periodontitis	46	Female	3.34	3.46	1.72	44.82
91	Periodontitis	64	Male	4.83	5.4	0.84	46.74
92	Periodontitis	34	Female	3.95	3.96	0.32	30.17
93	Periodontitis	55	Male	2.76	3.41	0.45	33.87
94	Periodontitis	57	Female	3.69	3.944	1.89	55.95
95	Periodontitis	55	Male	2.73	2.84	0.33	16.94
96	Periodontitis	51	Male	4.088	4.911	1.07	25
98	Periodontitis	47	Female	3.28	3.48	1.54	60.71
99	Periodontitis	48	Male	3.35	4.08	0.96	58.59
101	Periodontitis	45	Male	3.466	3.52	1.02	81
102	Periodontitis	39	Male	3.922	3.976	1.08	63.28
103	Periodontitis	53	Male	3.448	4.307	0.41	50.83
104	Periodontitis	60	Male	3.77	4	1.36	100
106	Periodontitis	48	Male	2.76	3.01	1.56	51.72
107	Periodontitis	41	Male	2.32	3.32	0.36	50.81
109	Periodontitis	55	Female	3.34	4.39	1	90.52
110	Periodontitis	57	Male	4.05	5.12	0.98	46.88
111	Periodontitis	36	Female	3.29	3.39	0.87	51.92
113	Periodontitis	54	Male	3.098	4.31	0.59	84.78
116	Periodontitis	57	Male	3.265	3.727	0.66	50

117	Periodontitis	57	Male	4.25	4.571	1.15	43.75
120	Healthy	32	Female	2.26	2.26	0.03	5.17
122	Healthy	23	Female	2.24	2.24	0.13	9.82
123	Healthy	24	Male	2.27	2.27	0.41	40
125	Healthy	23	Female	2.022	2.022	0.17	13.33
127	Healthy	24	Female	2.454	2.454	0	6.9
128	Healthy	19	Female	2.23	2.23	0.22	6.9
131	Healthy	23	Male	2.479	2.479	0.46	32.29
132	Healthy	27	Female	2.464	2.464	0.16	14.29
135	Healthy	36	Female	2.24	2.25	0.39	21.43
136	Healthy	22	Female	2.431	2.431	0.1	8.62
137	Healthy	31	Male	2.5	2.5	0.13	21.67
138	Healthy	28	Female	2.458	2.458	0.22	15.62
139	Healthy	24	Female	2.517	2.517	0.2	39.29
140	Healthy	41	Female	2.505	2.505	0.01	6.03
141	Healthy	25	Female	2.486	2.486	0.06	23
142	Periodontitis	42	Male	4.401	6	1.73	10
143	Periodontitis	53	Female	2.87	2.87	0.41	66.67
150	Periodontitis	50	Female	2.88	3.06	1.84	100
155	Periodontitis	58	Male	5.1	5.1	0.66	80
156	Healthy	21	Male	2.565	2.565	0	17.86
157	Healthy	36	Female	2.511	2.511	0.07	19.64
160	Healthy	26	Female	2.526	2.526	0.08	32
161	Periodontitis	37	Male	4.566	5.15	1.94	93.33
163	Periodontitis	40	Female	3.322	3.811	1.35	50
165	Periodontitis	54	Male	3.75	3.75	0.52	61.46
168	Periodontitis	49	Male	5.201	5.756	1.989	87.5
170	Periodontitis	63	Male	4.666	4.666	0.49	99.19
179	Periodontitis	62	Female	3.101	4.607	1.51	83.93
181	Periodontitis	54	Female	3.471	3.471	1.98	88.04
183	Periodontitis	86	Female	3.33	3.33	1.75	100
184	Periodontitis	85	Male	5.642	6.714	1.71	50
185	Periodontitis	72	Female	2.406	2.453	0.59	49.13
187	Periodontitis	34	Male	4.158	4.158	1.23	100
188	Periodontitis	64	Female	4.426	4.753	0.84	100
189	Periodontitis	62	Male	2.5	2.897	0.46	33.93
190	Periodontitis	58	Male	3.528	3.712	0.8	90
194	Periodontitis	58	Male	4.117	4.753	1.26	100
195	Periodontitis	37	Female	4.317	4.333	1.87	37.5
196	Periodontitis	62	Male	3.173	5.666	0.97	49.04
197	Periodontitis	41	Female	4.456	6.507	2.08	100
198	Periodontitis	36	Male	7.184	8.172	2.08	100
199	Periodontitis	59	Male	5.179	5.98	1.72	100
200	Periodontitis	52	Male	2.658	4.314	1.89	89.29
201	Periodontitis	49	Female	3.073	4.182	0.52	62.04
202	Periodontitis	62	Male	2.982	3.571	0.29	80.36
203	Periodontitis	40	Male	3.246	3.32	0.67	23.15
204	Periodontitis	47	Male	3.434	4.458	1.47	56.89
208	Periodontitis	64	Female	2.858	2.913	0.7	52.59
209	Periodontitis	56	Female	2.623	3.234	0.69	28.45
210	Periodontitis	65	Female	3.533	4.58	1.14	94.64
211	Periodontitis	48	Female	2.921	4.575	1.56	100
214	Periodontitis	72	Female	2.626	4.16	0.98	49.07
215	Periodontitis	43	Male	4.363	4.363	1.76	100
217	Periodontitis	57	Male	4.592	4.827	1.54	100
218	Periodontitis	53	Female	3.071	3.297	1.46	68.75
219	Periodontitis	36	Male	3.779	3.994	0.45	43.75
220	Periodontitis	54	Male	3.5	3.623	1.39	100
222	Periodontitis	58	Female	2.814	2.993	0.46	51.85

223	Healthy	28	Female	2.372	2.372	0.03	13.33
224	Healthy	34	Male	2.488	2.522	0.05	38.33
229	Periodontitis	74	Male	2.864	2.864	0.19	37.96
230	Periodontitis	63	Male	4.728	5.307	1.14	50
231	Periodontitis	60	Male	3.032	3.861	0.3	51.04
232	Periodontitis	44	Female	2.827	2.851	0.2	12.5
233	Periodontitis	65	Female	2.706	4.5	1.49	72.12
234	Periodontitis	65	Male	2.387	2.763	0.84	57.69
235	Periodontitis	48	Female	3.213	4.48	0.711	31.9
236	Periodontitis	55	Male	3.613	3.613	0.53	40.18
237	Periodontitis	54	Female	4.789	7.521	1.84	94.57
240	Periodontitis	47	Female	2.067	2.265	0.46	66.67
241	Periodontitis	46	Female	2.95	3.345	0.54	1.85
242	Periodontitis	64	Male	5.007	7.363	1.25	80.21
243	Periodontitis	36	Male	2.438	2.759	1.57	56.48
244	Periodontitis	50	Female	4.02	4.25	1.43	100
245	Periodontitis	39	Female	2.101	3.297	0.53	72.32
246	Periodontitis	47	Female	2.994	3.069	0.19	56.45
247	Periodontitis	48	Female	2.373	2.712	0.5	43.1
248	Healthy	21	Female	2.235	2.235	0.01	18.97
249	Periodontitis	59	Male	3.035	3.035	0.59	58.93
251	Periodontitis	70	Male	3.684	3.684	1.2	96.43
252	Periodontitis	35	Male	3.953	4.76	0.3	43
253	Periodontitis	79	Female	3.133	3.681	1.39	59.38
254	Periodontitis	47	Female	3.302	4.956	1.22	100
255	Periodontitis	57	Female	5.02	5.02	1.59	61.29
256	Periodontitis	38	Male	3.398	3.398	0.75	83.93
257	Periodontitis	47	Female	3.422	3.422	1	75
258	Periodontitis	64	Male	3.714	5	1.74	85.71
259	Periodontitis	56	Male	3.38	4.833	1.38	49.07
260	Healthy	31	Female	2.438	2.438	0.03	10
263	Periodontitis	44	Male	3.61	4.96	0.64	54.17
267	Periodontitis	37	Female	6.133	6.133	2	100
268	Periodontitis	56	Male	4.911	4.911	1.48	89.17
269	Periodontitis	45	Female	3.994	4.529	0.32	66.07
271	Periodontitis	76	Female	4.285	4.968	1.21	61.54
272	Periodontitis	64	Male	4.302	4.703	1.64	100
273	Periodontitis	64	Male	3.267	3.857	0.83	52.68
274	Periodontitis	30	Male	2.309	2.886	1.13	95.54
275	Periodontitis	50	Female	4.345	4.607	1.05	80.35
277	Periodontitis	39	Male	3.307	3.679	0.77	54.8
278	Periodontitis	55	Male	4.129	5.29	0.46	34.26
279	Periodontitis	54	Male	5.453	7.4	1.96	80.77
280	Periodontitis	69	Female	2.897	2.897	0.14	42.86
281	Periodontitis	58	Male	2.694	4.243	1.31	67.7
282	Periodontitis	38	Female	2.719	2.719	0.82	75
284	Periodontitis	81	Female	2.944	2.944	0.19	23.96
287	Periodontitis	60	Female	3.533	3.766	0.63	35.42
296	Periodontitis	40	Female	5.724	5.724	1.2	43.1
297	Periodontitis	59	Male	3.617	4.253	0.48	60
300	Periodontitis	28	Female	4.428	4.726	0.62	55.36
302	Periodontitis	47	Female	3.416	3.416	1.38	100
303	Periodontitis	51	Male	3.488	3.94	0.7	92.86
306	Healthy	21	Female	2.472	2.472	0.17	23
310	Periodontitis	42	Male	3.897	4.064	1.71	89.17
314	Periodontitis	52	Female	3.994	4.244	0.36	66.67
315	Healthy	24	Male	2.27	2.27	0.22	31.89
316	Periodontitis	59	Female	2.386	2.41	0.64	62.5
317	Periodontitis	42	Male	5.135	6.277	1.59	71.3

318	Healthy	27	Female	2.255	2.255	0.14	16.07
319	Healthy	23	Male	2.297	2.297	0.03	9.82
320	Healthy	31	Male	2.321	2.344	0	43.97
321	Healthy	30	Female	2.436	2.436	0.03	8.62
322	Periodontitis	64	Female	3.944	4.141	0.62	65.74
323	Healthy	25	Male	2.422	2.422	0.05	28.12
324	Periodontitis	39	Male	6.686	8.66	1.14	100
325	Periodontitis	72	Male	2.814	4.49	0.68	20.54
326	Healthy	23	Male	2.442	2.442	0.21	18.97
329	Healthy	18	Female	2.422	2.422	0.11	23.21
331	Healthy	25	Male	2.512	2.512	0.02	2.78
332	Healthy	31	Male	2.521	2.521	0.06	13.39
333	Healthy	35	Male	2.624	2.624	0.12	8.33
335	Periodontitis	65	Female	3.88	4.803	1.21	55.36
336	Healthy	32	Female	2.563	2.563	0.04	1.72
337	Healthy	25	Male	2.604	2.604	0.03	5.46
338	Healthy	16	Female	2.625	2.625	0.28	20.83
339	Healthy	31	Female	2.706	2.706	0.31	26.72
340	Healthy	22	Male	2.621	2.621	0.12	5
342	Periodontitis	77	Female	3.366	3.64	0.52	100
343	Periodontitis	54	Male	3.522	3.672	1.17	91.38
344	Periodontitis	54	Male	3.886	3.933	1.32	66.96
345	Periodontitis	69	Male	2.568	2.839	0.28	64.17
346	Periodontitis	64	Male	5.363	5.654	1.77	34.82
347	Healthy	28	Male	2.526	2.526	0.14	10.16
350	Periodontitis	55	Male	3.587	5.277	1.05	75
352	Healthy	26	Female	2.482	2.482	0.24	33.03
354	Periodontitis	57	Male	3.06	3.06	0.47	62.96
355	Periodontitis	64	Female	2.638	3.055	0.15	66.3
357	Periodontitis	54	Male	3.48	3.76	1.44	54
358	Periodontitis	68	Female	3.919	3.919	1.06	52.62
360	Healthy	27	Male	2.408	2.408	0.01	4.31
361	Healthy	21	Male	2.471	2.471	0.41	7.76
362	Healthy	33	Male	2.586	2.586	0.22	25
363	Healthy	39	Male	2.577	2.577	0.25	15.83
364	Healthy	40	Male	2.591	2.591	0.09	6.03
365	Periodontitis	58	Female	3.505	4.583	0.63	63.93
367	Periodontitis	53	Male	3.794	3.805	0.57	45.83
368	Periodontitis	55	Female	4.281	5.34	1.45	56.73
371	Periodontitis	52	Female	2.119	2.19	0.56	60.71
372	Periodontitis	64	Male	2.252	2.591	0.47	48.28
373	Healthy	24	Female	2.369	2.369	0.07	3.57
376	Healthy	33	Female	2.541	2.541	0.01	0
377	Healthy	21	Male	2.511	2.511	0.09	7
378	Healthy	21	Female	2.235	2.235	0.02	18.97
379	Healthy	22	Female	2.62	2.62	0.07	2.59
381	Healthy	25	Female	2.619	2.619	0.28	32.14
384	Healthy	33	Female	2.47	2.47	0.008	1.79
385	Periodontitis	61	Female	4.039	4.039	1.04	78
387	Periodontitis	66	Female	4.875	5.416	0.63	77.78
388	Healthy	20	Female	2.326	2.326	0.04	20.83
391	Periodontitis	62	Female	2.791	3.91	0.85	79.31

Table S3. Summary of the correlation analysis of bacterial species with demographic characteristics and clinical parameters.

Bacterial species	Demographic characteristics/ clinical parameters	Pearson correlation	P-value
Bacteroidetes_[G-3] bacterium_HMT_280	PD	0.306	2.72E-13
Porphyromonas gingivalis	Age	0.351	3.37E-17
Porphyromonas gingivalis	PD	0.382	2.17E-20
Porphyromonas gingivalis	CAL	0.406	5.42E-23
Porphyromonas gingivalis	GI	0.317	3.85E-14
Porphyromonas gingivalis	PI	0.303	4.77E-13
Tannerella forsythia	PD	0.324	9.66E-15
Tannerella forsythia	CAL	0.349	5.00E-17
Prevotella sp._HMT_304	CAL	0.335	9.79E-16
Peptoniphilaceae_[G-1] bacterium_HMT_113	Age	0.321	1.73E-14
Filifactor alocis	PD	0.336	8.03E-16
Filifactor alocis	CAL	0.351	3.30E-17
Peptostreptococcaceae_[XI][G-5] saphenum	CAL	0.305	3.87E-13
Peptostreptococcaceae_[XI][G-6] nodatum	PD	0.345	1.26E-16
Peptostreptococcaceae_[XI][G-6] nodatum	CAL	0.375	1.28E-19
Peptostreptococcaceae_[XI][G-6] nodatum	GI	0.404	8.74E-23
Peptostreptococcaceae_[XI][G-6] nodatum	PI	0.327	5.45E-15
Mycoplasma faucium	PD	0.360	4.72E-18
Mycoplasma faucium	CAL	0.383	1.90E-20
Mycoplasma faucium	GI	0.303	5.20E-13
Desulfobulbus sp._HMT_041	PD	0.314	5.99E-14
Desulfobulbus sp._HMT_041	CAL	0.325	7.50E-15
Treponema denticola	PD	0.340	3.14E-16
Treponema denticola	CAL	0.339	4.38E-16
Treponema denticola	GI	0.303	5.33E-13
Fretibacterium fastidiosum	PD	0.320	2.12E-14
Fretibacterium fastidiosum	CAL	0.308	1.99E-13

Table S4. Differential abundance of bacterial species using DESeq2 method.

Bacterial species	Sampling site	Log_2(Fold Change)	adjusted p-value
**Dominant bacterial species in the periodontitis group**			
Actinomyces cardiffensis	Buccal	9.261	1.09E-06
Peptidiphaga gingivicola	Buccal	3.918	0.00012209
Bacteroidetes_[G-3] bacterium_HMT_280	Buccal	6.100	3.14E-06
Bacteroidetes_[G-6] bacterium_HMT_516	Buccal	19.186	0.000119455
Bacteroidaceae_[G-1] bacterium_HMT_272	Buccal	7.651	1.26E-06
Porphyromonas endodontalis	Buccal	4.242	4.68E-07
Porphyromonas gingivalis	Buccal	9.444	9.34E-13
Tannerella forsythia	Buccal	5.445	5.25E-10
Prevotella dentalis	Buccal	4.855	3.19E-05
Prevotella sp._HMT_304	Buccal	10.910	4.85E-08
Prevotella sp._HMT_443	Buccal	10.142	0.000303763
Prevotella sp._HMT_526	Buccal	20.538	2.36E-13
Anaerolineae_[G-1] bacterium_HMT_439	Buccal	8.706	4.36E-08
Gemella haemolysans	Buccal	2.641	0.000567746
Streptococcus constellatus	Buccal	4.751	0.000108299
Clostridiales_[F-1][G-1] bacterium_HMT_093	Buccal	17.057	3.00E-12
Johnsonella sp._HMT_166	Buccal	7.592	2.91E-05
Lachnospiraceae_[G-8] bacterium_HMT_500	Buccal	6.754	3.74E-08
Stomatobaculum sp._HMT_373	Buccal	29.147	7.55E-13
Peptoniphilaceae_[G-1] bacterium_HMT_113	Buccal	27.187	5.42E-11
Filifactor alocis	Buccal	8.476	1.04E-12
Mogibacterium timidum	Buccal	7.153	1.43E-06
Peptostreptococcaceae_[XI][G-5] saphenum	Buccal	22.320	8.38E-14
Peptostreptococcaceae_[XI][G-6] minutum	Buccal	5.403	0.000127189
Peptostreptococcaceae_[XI][G-6] nodatum	Buccal	14.157	8.21E-19
Mollicutes_[G-2] bacterium_HMT_906	Buccal	6.514	0.000794244
Mycoplasma faucium	Buccal	8.732	3.74E-09
Fusobacterium nucleatum_subsp._vincentii	Buccal	3.302	0.0001013
Fusobacterium sp._HMT_203	Buccal	53.438	8.81E-06
Desulfobulbus sp._HMT_041	Buccal	12.266	1.02E-11
Aggregatibacter aphrophilus	Buccal	3.770	0.00012209
Treponema denticola	Buccal	5.527	1.62E-08
Treponema maltophilum	Buccal	3.728	8.26E-05
Treponema sp._HMT_237	Buccal	3.423	0.000290301
Treponema sp._HMT_257	Buccal	21.890	1.04E-09
Treponema sp._HMT_258	Buccal	7.033	0.000247879
Treponema sp._HMT_927	Buccal	32.511	9.13E-08
Fretibacterium fastidiosum	Buccal	4.264	1.47E-06
Fretibacterium sp._HMT_362	Buccal	19.345	6.51E-09
Actinomyces cardiffensis	Supragingival	8.726	0.000898727
Actinomyces israelii	Supragingival	4.049	0.000528663
Bacteroidetes_[G-3] bacterium_HMT_280	Supragingival	11.062	7.61E-06
Bacteroidaceae_[G-1] bacterium_HMT_272	Supragingival	6.789	0.000537702
Porphyromonas gingivalis	Supragingival	12.897	1.08E-10
Alloprevotella rava	Supragingival	25.365	1.17E-06
Prevotella intermedia	Supragingival	4.499	0.000995149
Prevotella melaninogenica	Supragingival	6.931	1.96E-06
Prevotella sp._HMT_304	Supragingival	7.524	0.000475656
Prevotella sp._HMT_443	Supragingival	27.035	0.000231073
Prevotella sp._HMT_526	Supragingival	16.069	1.82E-05
Capnocytophaga sp._HMT_332	Supragingival	25.199	3.97E-06
Capnocytophaga sp._HMT_863	Supragingival	21.591	7.61E-06
Capnocytophaga sputigena	Supragingival	3.016	0.000513803
Anaerolineae_[G-1] bacterium_HMT_439	Supragingival	26.260	9.54E-13
Abiotrophia defectiva	Supragingival	8.397	3.61E-05
Streptococcus constellatus	Supragingival	8.477	2.36E-05
Clostridiales_[F-1][G-1] bacterium_HMT_093	Supragingival	16.816	7.61E-06
Pseudoramibacter alactolyticus	Supragingival	17.233	0.000155577
Catonella sp._HMT_451	Supragingival	26.322	9.56E-07
Johnsonella sp._HMT_166	Supragingival	17.613	3.24E-06
Stomatobaculum sp._HMT_373	Supragingival	40.116	1.32E-08
Peptoniphilaceae_[G-1] bacterium_HMT_113	Supragingival	77.033	1.29E-11

Filifactor alocis	Supragingival	5.898	8.26E-06
Peptostreptococcaceae_[XI][G-1] bacterium_HMT_383	Supragingival	15.983	0.000982254
Peptostreptococcaceae_[XI][G-5] saphenum	Supragingival	28.574	2.49E-10
Peptostreptococcaceae_[XI][G-6] nodatum	Supragingival	13.793	2.49E-10
Peptostreptococcus stomatis	Supragingival	3.759	0.000128675
Mollicutes_[G-2] bacterium_HMT_906	Supragingival	23.079	0.000898727
Mycoplasma faucium	Supragingival	7.804	6.52E-06
Selenomonas dianae	Supragingival	12.278	1.01E-06
Selenomonas sp._HMT_481	Supragingival	5.638	0.000155577
Dialister invisus	Supragingival	3.937	0.000344528
Leptotrichia wadei	Supragingival	6.496	5.10E-05
Sneathia sanguinegens	Supragingival	58.092	0.000140285
Gracilibacteria_(GN02)_[G-2] bacterium_HMT_873	Supragingival	34.400	4.47E-07
Ottowia sp._HMT_894	Supragingival	5.014	0.000393987
Neisseria flavescens	Supragingival	8.530	0.000160547
Campylobacter rectus	Supragingival	2.890	0.000132239
Treponema amylovorum	Supragingival	40.339	7.26E-07
Treponema denticola	Supragingival	5.838	6.52E-06
Treponema sp._HMT_258	Supragingival	52.576	8.21E-09
Fretibacterium fastidiosum	Supragingival	5.871	1.09E-05
**Dominant bacterial species in the healthy group**			
Actinomyces gerencseriae	Buccal	5.088	0.000247879
Actinomyces sp._HMT_180	Buccal	2.606	0.000423873
Corynebacterium durum	Buccal	3.139	6.38E-05
Cutibacterium acnes	Buccal	8.082	1.72E-06
Prevotella nanceiensis	Buccal	3.523	0.000241981
Prevotella oulorum	Buccal	4.342	0.000200781
Enterococcus faecalis	Buccal	63.097	5.07E-06
Oribacterium asaccharolyticum	Buccal	4.265	0.000709683
Leptotrichia sp._HMT_223	Buccal	10.747	0.000423873
Haemophilus parainfluenzae	Buccal	2.347	0.00096722
Saccharibacteria_(TM7)_[G-6] bacterium_HMT_870	Buccal	7.531	0.000686501
Actinomyces massiliensis	Supragingival	3.563	1.82E-05

Table S5. Evaluation of prediction models using four feature sets and five machine learning algorithms.

Feature set	Site	Algorithm	Accuracy	F_1 score	Sensitivity	Specificity	AUC[1]
ALL	Buccal	KNN[2]	0.875	0.920	0.933	0.677	0.900
ALL	Buccal	LMT[3]	0.893	0.931	0.938	0.742	0.919
ALL	Buccal	LogitBoost	0.879	0.923	0.938	0.677	0.896
ALL	Buccal	SMO[4]	0.853	0.910	0.967	0.468	0.717
ALL	Buccal	Naïve Bayes	0.868	0.911	0.876	0.839	0.897
DESeq2	Buccal	KNN	0.879	0.922	0.924	0.726	0.904
DESeq2	Buccal	LMT	0.879	0.922	0.929	0.710	0.902
DESeq2	Buccal	LogitBoost	0.871	0.918	0.929	0.677	0.890
DESeq2	Buccal	SMO	0.882	0.926	0.957	0.629	0.793
DESeq2	Buccal	Naïve Bayes	0.864	0.908	0.871	0.839	0.909
LefSe	Buccal	KNN	0.875	0.920	0.933	0.677	0.872
LefSe	Buccal	LMT	0.835	0.894	0.900	0.613	0.861
LefSe	Buccal	LogitBoost	0.831	0.893	0.914	0.548	0.797
LefSe	Buccal	SMO	0.853	0.907	0.933	0.581	0.765
LefSe	Buccal	Naïve Bayes	0.835	0.888	0.848	0.790	0.881
DESeq2+LefSe	Buccal	KNN	0.886	0.926	0.924	0.758	0.910
DESeq2+LefSe	Buccal	LMT	0.875	0.919	0.924	0.710	0.909
DESeq2+LefSe	Buccal	LogitBoost	0.864	0.912	0.914	0.694	0.880
DESeq2+LefSe	Buccal	SMO	0.879	0.924	0.957	0.613	0.785
DESeq2+LefSe	Buccal	Naïve Bayes	0.853	0.900	0.857	0.839	0.904
ALL	Supragingival	KNN	0.813	0.877	0.862	0.645	0.791
ALL	Supragingival	LMT	0.820	0.884	0.886	0.597	0.853
ALL	Supragingival	LogitBoost	0.838	0.899	0.929	0.532	0.858
ALL	Supragingival	SMO	0.805	0.883	0.952	0.306	0.629
ALL	Supragingival	Naïve Bayes	0.728	0.805	0.729	0.726	0.793
DESeq2	Supragingival	KNN	0.757	0.829	0.762	0.742	0.745
DESeq2	Supragingival	LMT	0.801	0.874	0.895	0.484	0.835
DESeq2	Supragingival	LogitBoost	0.853	0.907	0.933	0.581	0.895
DESeq2	Supragingival	SMO	0.835	0.899	0.952	0.435	0.694
DESeq2	Supragingival	Naïve Bayes	0.750	0.816	0.719	0.855	0.853
LefSe	Supragingival	KNN	0.813	0.881	0.895	0.532	0.789
LefSe	Supragingival	LMT	0.783	0.865	0.900	0.387	0.798
LefSe	Supragingival	LogitBoost	0.846	0.907	0.971	0.419	0.813
LefSe	Supragingival	SMO	0.809	0.883	0.938	0.371	0.657
LefSe	Supragingival	Naïve Bayes	0.717	0.801	0.738	0.645	0.769
DESeq2+LefSe	Supragingival	KNN	0.801	0.864	0.819	0.742	0.845
DESeq2+LefSe	Supragingival	LMT	0.783	0.862	0.876	0.468	0.827
DESeq2+LefSe	Supragingival	LogitBoost	0.853	0.905	0.905	0.677	0.873
DESeq2+LefSe	Supragingival	SMO	0.835	0.900	0.962	0.413	0.691
DESeq2+LefSe	Supragingival	Naïve Bayes	0.732	0.806	0.724	0.758	0.836

AUC[1]: Area under the ROC curve; KNN[2]: K nearest neighbor; LMT[3]: logistic model tree; SMO[4]: SVMs with sequential minimal optimization.

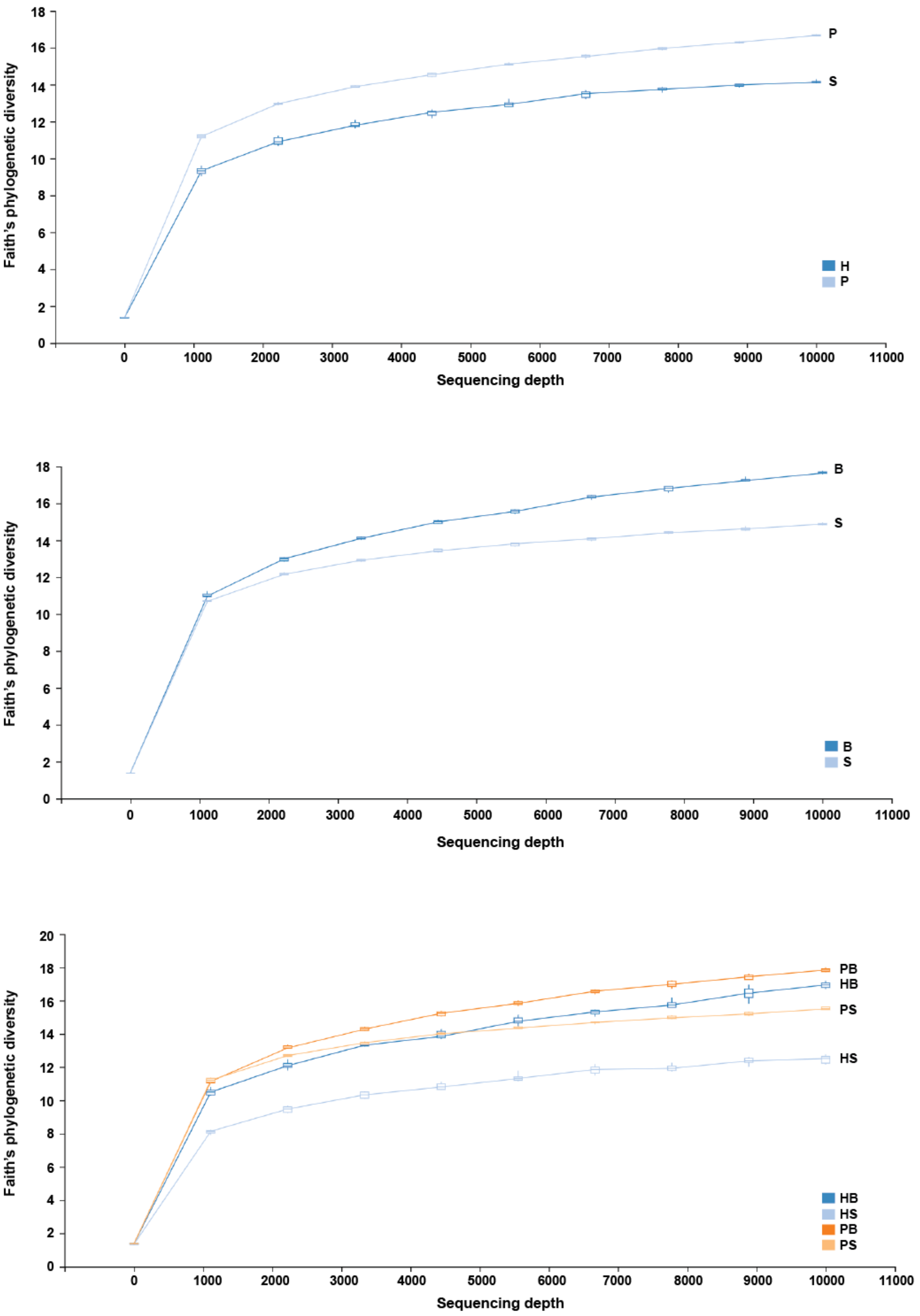

Figure 1. Rarefaction curves of community diversity (faith's phylogenetic diversity) for the disease status; healthy (H) and periodontitis (P), sample sites; buccal mucosa (B) and supragingival space (S) and the four subject groups; HB, HS, PB and PS.

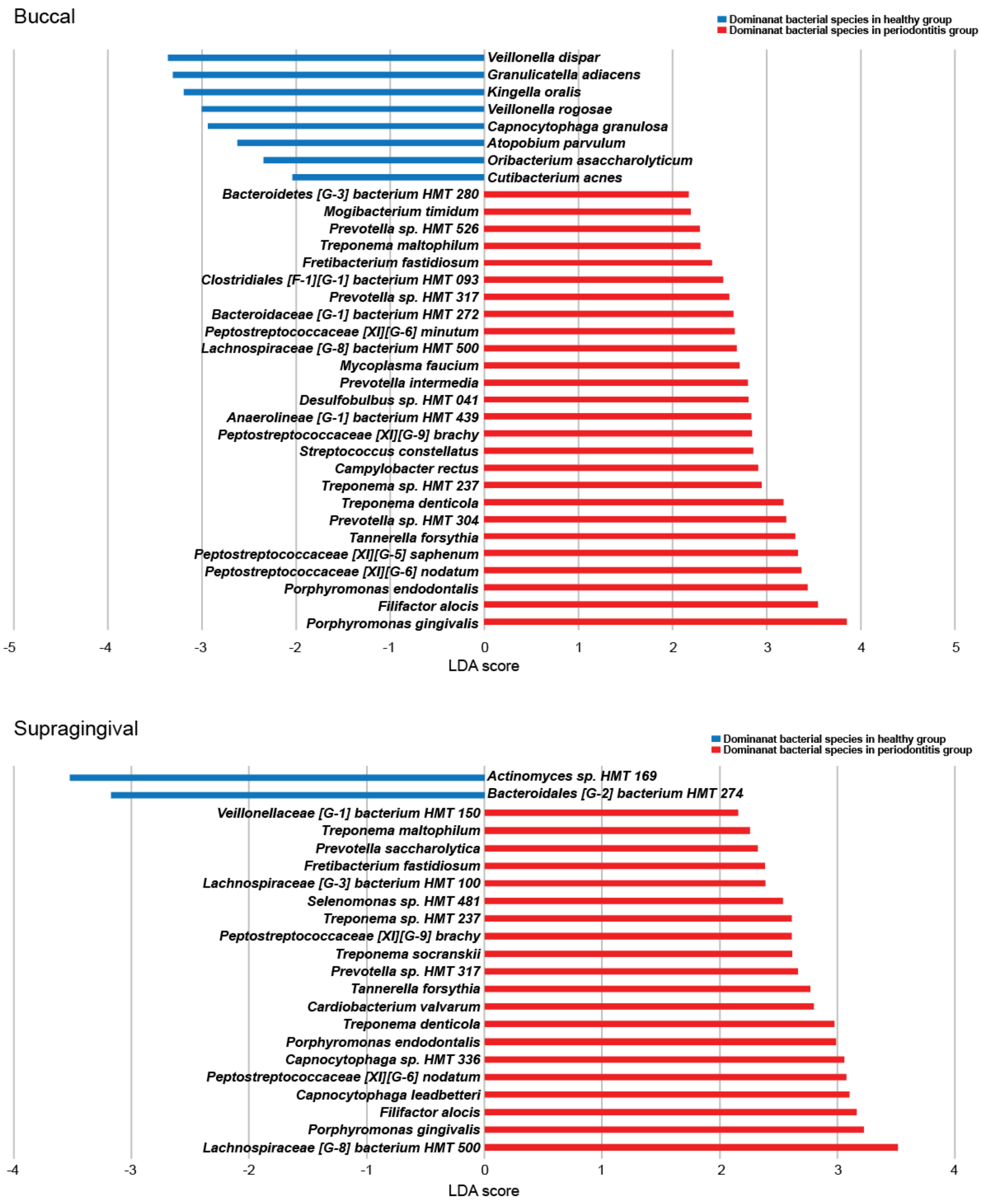

Figure 2. Differential abundance analysis of bacterial species by LefSe method.
